# A Text-Book of Comparative Physiology

**Published:** 1890-12

**Authors:** 


A TEXT-BOOK OF COMPARATIVE PHYSIOLOGY. BY WESLEY MILLS, M. D., D. V. S., Professor of Human and Comparative Physiology in McGill University, Montreal. D. Appleton & Co., New York, 1890, pp. 636.

This is an abridgment of the author's larger work on Animal Physiology, embodying the same plan, and is designed to meet the requirements of students and practitioners of comparative, or veterinary medicine. Comparative medicine is now recognized as having a fixed place in medical science. Heretofore, the writer tells us too little has been done to instruct the English-speaking student in the important principles of veterinary science, and therefore Dr. Mills' book will supply a real want.

In general biology we note that the author strongly advocates the kinship of man to the lower animals, and assumes that the doctrine of organic evolution has now become a great scientific principle.

The book is complete and well written; and the author's style is easy, but forcible. In the matter of illustrations, which are numerous, and in general workmanship the publishers have done their work in a manner that leaves nothing to be desired.

It gives us pleasure to commend the work as a whole; and particularly the sections on the brain, and embryology, as being an exposition of our latest knowledge in these important departments of animal physiology. L. B. G.

